# Cobalt(III)–Schiff Base Coordination to Nsp1, a SARS-CoV‑2 Protein

**DOI:** 10.1021/acs.inorgchem.6c01698

**Published:** 2026-07-22

**Authors:** Maryann Morales, Moon Young Yang, William A. Goddard, Harry B. Gray, Jay R. Winkler

**Affiliations:** 6469 Beckman Institute, California Institute of Technology, Pasadena, California 91125, United States

## Abstract

Severe acute respiratory syndrome coronavirus 2 (SARS-CoV-2) suppresses host immune defenses through the activity of nonstructural protein 1 (Nsp1), which inhibits host mRNA translation by occluding the mRNA entry channel of the 40S ribosomal subunit. Here, we investigate whether Co­(III)­(acacen)­(NH_3_)_2_^+^ (acacen = bis­(acetylacetone)­ethylenediamine) can disrupt Nsp1 function through coordination to histidine residues. Using ^59^Co nuclear magnetic resonance (NMR) spectroscopy, we show that Co­(III)­(acacen)­(NH_3_)_2_^+^ undergoes axial ligand substitution upon interaction with an Nsp1-derived peptide encompassing the functionally critical His165 residue. Computational analysis supports a coordination structure in which an axial NH_3_ ligand is displaced by a histidine imidazole. Although proteolytic fragmentation followed by mass spectrometric analysis revealed multiple histidine binding sites in full-length Nsp1, assigned to His13, the His81/His83 region, and His165, *in vitro* translation assays confirmed that the addition of Co­(III)­(acacen)­(NH_3_)_2_^+^ did not restore host protein synthesis.

## Graphical abstract

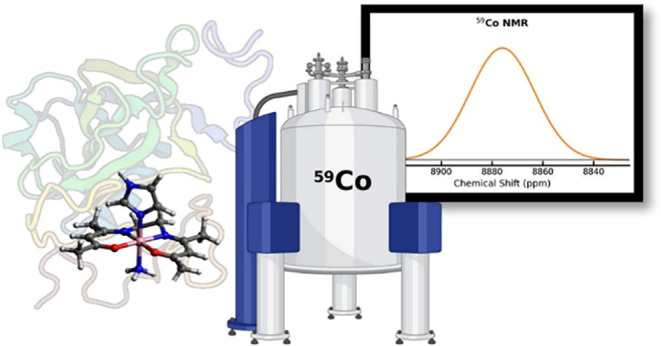

## Introduction

A central factor contributing to the pathogenicity and persistence of SARS-CoV-2 is its capacity to evade and suppress host immune defenses through a variety of mechanisms. These include shielding viral replication intermediates, mimicking host molecular structures, disrupting antiviral signaling cascades, and weakening interferon responses.

One prominent mechanism is inhibition of protein synthesis, in which the virus impairs host mRNA translation. In the process of blocking the production of cellular proteins, SARS-CoV-2 both redirects the translational machinery toward viral RNA and suppresses the host’s ability to mount an antiviral response.^1^ A key component of this process is nonstructural protein 1 (Nsp1), a conserved coronavirus factor that inhibits host mRNA translation.^2,3^

During SARS-CoV-2 infection, the overlapping open reading frame ORF1ab is translated from the positive-sense genomic RNA into a large polyprotein that undergoes autoproteolysis to create 16 nonstructural proteins (Nsps), the first of which is Nsp1.^4^ Nsp1 employs a multipronged strategy to suppress host protein synthesis, with inhibition of translation primarily attributable to its interaction with the 40S ribosomal subunit.^1,2,5^ Upon binding, the intrinsically disordered C-terminal domain of Nsp1 adopts a helix-turn-helix conformation that occludes the mRNA entry channel, thereby blocking translation initiation and promoting degradation of host mRNAs.^3,6–8^ This interaction is critically mediated by a short loop connecting the two helices, which contains residues Lys164 and His165. Structural and mutational studies have shown that Lys164 and His165 engage the 18S rRNA through stacking and backbone interactions, and disruption of these residues abolishes Nsp1-mediated translation inhibition, mRNA degradation, and suppression of type I interferon responses.^8,9^ These findings underscore the functional importance of the Lys164-His165 region and highlight it as a key determinant of Nsp1 pathogenic activity.

Together, these interactions highlight the C-terminal domain of Nsp1 as a crucial regulator of the host response to coronavirus infection. Given its central role in protein synthesis inhibition, this region is an attractive therapeutic target: disrupting Nsp1–40S ribosome binding could restore host protein synthesis and immune signaling, therefore improving antiviral defense.

Investigators have computationally screened libraries of commercially available organic drugs in the search of candidates that might target Nsp1.^10^ While some hits have been identified, their binding affinities are insufficient to produce meaningful therapeutic effects. This has prompted exploration beyond traditional organic molecules toward metal-based complexes, which offer well-defined three-dimensional structures and access to novel modes of action not always available in organic compounds.

Medicine has advanced substantially over time, with therapeutic strategies evolving from an exclusive reliance on organic molecules to the incorporation of metal-based complexes. Cisplatin was first synthesized in 1845 by Michele Peyrone, but its biological activity was not recognized until 1965 through the work of Barnett Rosenberg.^11,12^ This discovery revolutionized medicine by opening an entirely new field focused on the therapeutic properties of metal complexes.^13–15^ As a result, metal-containing reagents have emerged as a distinct class of drug candidates.^16^

We have exploited the sensitivity of intrinsically disordered Nsp1 regions to metal-ion coordination. Transition metal ions can bind N-donor and O-donor side chains, altering protein conformation and function. In previous work we demonstrated that copper­(II) binds to the disordered C-terminal region of Nsp1 by His165 imidazole and backbone ligation,^17^ although the rapid ligand exchange properties of this complex disfavor therapeutic applications.^17,18^ We think it likely that metal ions that form substitution-inert complexes will be better at selectively attacking viral proteins.

Cobalt­(III) Schiff-base complexes exhibit antiviral activity, and their interactions with both proteins and nucleic acids have been studied extensively.^19,20^ In viral proteins, binding to the Co­(III) center often occurs by substitution of histidine imidazole for an axial ammine or other ligand. The robust Co­(III) coordination framework provides a highly tunable platform, allowing for precise control over both axial and equatorial ligation.^19,21^

In this work, we investigated Co­(III)­(acacen)­(NH_3_)_2_^+^ interactions with Nsp1-CT_10_, a C-terminal 10 residue peptide (Figure 1), since an imidazole can displace an ammine in an axial position of this complex.^19,20,22,23^ We employed ^59^Co NMR and other spectroscopic techniques along with quantum mechanics (QM) calculations to monitor changes in the Co­(III) coordination environment in reactions with Nsp1-CT_10_. In addition, we used proteolytic fragmentation of full-length Nsp1 coupled with mass spectrometry to identify histidine residues bound to Co­(III); and we evaluated the inhibitory capacity of Co­(III) using *in vitro* translation assays.

**Figure 1:**
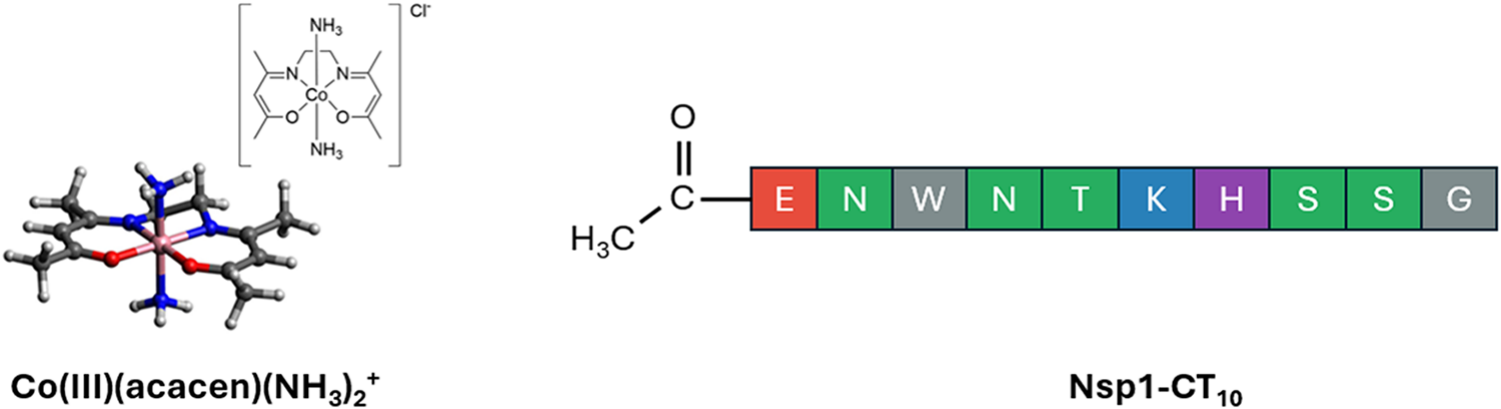
Left: Drawing of Co­(III)­(acacen)­(NH_3_)_2_^+^; Right: Nsp1-CT_10_ sequence (acetylated N-terminus).

## Methods

### Decapeptide

N-terminally acetylated decapeptide Ac-ENWNTKHSSG (Nsp1-CT_10_) was obtained from Genscript Biotech Corporation.

### Full-Length Nsp1

The following procedure was adapted from previous literature.^3^

Plasmids encoding the WT Nsp1 sequence were obtained from GenScript Biotech. The plasmid was transformed into *E. coli* BL21­(DE3) and cells were grown in 2xYT medium at 37 °C. When the cultures reached an optical density at 600 nm (OD_600_) of 0.8, IPTG was added to a final concentration of 0.5 mM and the temperature was lowered to 18 °C. Cells were harvested after 16 h by centrifugation (4000 rpm, 10 min, 4 °C). Pellets were resuspended in lysis buffer (50 mM HEPES-KOH pH 7.6, 500 mM KCl, 5 mM MgCl_2_, 10% (wt/vol) glycerol, 0.5 mM TCEP) and lysed using a sonicating tip (80% amplitude, 5 min, 3 s on, 17 s off). The lysed cells were stirred with DNase, RNase, and protease inhibitor for an hour before clearing by centrifugation (12,000 rpm, 30 min, 4 °C). The lysate was loaded onto a Ni-NTA resin column, then the protein was washed with the lysis buffer containing increasing concentrations of imidazole up to 40 mM. The His_6_-tagged protein was eluted with lysis buffer containing 300 mM imidazole (Im). The eluent was buffer-exchanged into lysis buffer to remove imidazole. The samples were incubated with TEV protease (NEB # P8112). The cleaved Nsp1 was isolated on a Ni-NTA resin column. The sample was buffer-exchanged into its storage buffer (40 mM HEPES-KOH pH 7.6, 200 mM KCl, 40 mM MgCl_2_, 10% (wt/vol) glycerol, 1 mM TCEP) and concentrated using an Amicon Ultra-15 centrifugal filter (3-kDA molecular weight cutoff (MWCO)), then flash frozen in liquid nitrogen and stored at −80 °C.

**Scheme 1:**
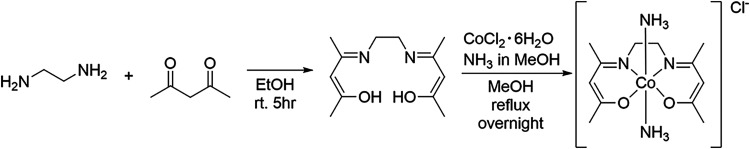
Scheme for Acacen and Co­(III)­(acacen)­(NH_3_)_2_^+^ Synthesis

### Synthetic Methods

Synthesis was based on a literature procedure (Scheme 1).^22^ Co­(III)­(acacen)­(NH_3_)_2_^+^ is a known compound; characterization data reported in the SI are consistent with those in a previous report.^20^

#### Ligand Synthesis

Ethylenediamine (2.71 g, 45.1 mmol) was dissolved in ethanol (50 mL). A solution of freshly distilled acetylacetone (9.21 mL, 90.2 mmol) in ethanol was added dropwise over 2 h with continuous stirring. The mixture was stirred for an additional 3 h, then filtered. The solid was washed with water (3 × 20 mL) and diethyl ether (3 × 20 mL) and dried under vacuum to afford product (acacen).

^1^H NMR (400 MHz, CDCl_3_) δ 10.90 (s, 2H), 4.99 (s, 2H), 3.49–3.36 (m, 4H), 2.00 (s, 6H), 1.91 (s, 6H).

#### [Co­(acacen)­(NH_3_)_2_]­Cl

After acacen (500 mg, 2.22 mmol) was dissolved in methanol (50 mL), cobalt­(II) chloride hexahydrate (541 mg, 2.27 mmol) was added under a nitrogen atmosphere, and the solution was heated to 60 °C with stirring for 30 min. Saturated ammonia in methanol (0.25 mL) was introduced, and the mixture was refluxed for 1 h. More (2.5 mL) saturated ammonia in methanol was added, after which oxygen was bubbled through the reaction mixture overnight. The solid product was collected by filtration, washed with cold methanol (3 × 10 mL) followed by diethyl ether (3 × 10 mL), and dried under vacuum.

^1^H NMR assignments are in the SI.

### Spectroscopic Methods

#### ^59^Co NMR Spectra

NMR spectra were recorded in D_2_O solutions. A Bruker Neo 400 with a broadband iProbe operating at 95.79 MHz was used to obtain ^59^Co NMR spectra. Standard parameters: 16,000 scans with a 10-microsecond pulse, 20 ms acquisition time, 100 ms relaxation delay, and a 100,000 Hz (1044 ppm) sweep width.

^59^Co NMR spectra were collected in D_2_O solutions to characterize the cobalt coordination environment before and after reaction with Nsp1-CT_10_. Nsp1-CT_10_ was added to a D_2_O solution of Co­(acacen)­(NH_3_)_2_Cl until a 1:1 reaction mixture was obtained, and solutions were incubated overnight at room temperature to allow for ligand exchange. The overnight equilibration period also ensured complete deuterium exchange at the axial ammine ligands that can otherwise contribute to spectral heterogeneity and line broadening in D_2_O.^24–27^ Spectra were acquired on 10 mM Co­(III):Nsp1-CT_10_ solutions.

The reference standard for ^59^ Co NMR spectra was Kc_3_[Co­(CN)_6_] (set to 0 ppm). The ^59^Co NMR spectrum of the standard (20 mM in D_2_O) is in the repository.

#### Mass Spectrometry

##### Sample Preparation

Purified protein samples were dissolved in 8 M urea (Sigma) buffered with 50 mM HEPES (pH 7.5). The samples were reduced using 5 mM tris­(2-carboxyethyl)­phosphine (TCEP, Thermo Fisher Scientific) and alkylated using 20 mM chloroacetamide (CAA, Sigma). The samples were diluted using 50 mM HEPES (pH 7.5) until the urea concentration was lower than 2 M, and CaCl_2_ (Sigma, 5 mM) was added. Trypsin was added to the sample with a 1:50 enzyme to substrate ratio according to protein quantitation, followed by digestion at 37 °C overnight (∼14 h).

After trypsinization, the digested peptides were eluted from the S-trap device according to the manufacturer’s protocol and dried using a speedvac. The samples were subsequently desalted again using Pierce C18 Spin Columns (Thermo Fisher Scientific, 89870) according to the manufacturer’s protocol and dried again. The dried samples were stored at −80° before LC-MS/MS analysis.

##### LC-MS/MS Analysis

Samples were resuspended in 0.1% formic acid (Thermo Fisher Scientific, 85178) solution, and peptide concentration was tested using the Pierce Quantitative Fluorometric Peptide Assay (Thermo Fisher Scientific, 23290). LC-MS/MS experiments were performed by loading a 500 μg sample onto a Thermo Vanquish Neo UHPLC system (Thermo Fisher Scientific) connected to an Orbitrap Eclipse Tribrid mass spectrometer (Thermo Fisher Scientific). Peptides were separated on an Aurora UHPLC Column (25 cm × 75 μm, 1.6 μm C18, AUR2–25075C18A, Ion Opticks) with a flow rate of 0.35 mL/min for 70 min. The gradient was composed of 2–6% solvent B for 3.5 min, 6 to 25% B for 41.5 min, 25 to 40% B for 15 min, 40 to 98% B for 2 min, 98% B for 5 min, and 98 to 2% B for 3 min. Solvent A was 97.8% H_2_O, 2% acetonitrile, and 0.2% formic acid, and solvent B was 19.8% H_2_O, 80% acetonitrile, and 0.2% formic acid. MS1 scans were acquired in the range 375 to 1600 *m*/*z* in the Orbitrap at 120 k resolution. The maximum injection time was 50 ms, and the automatic gain control target was 250%. MS2 scans were acquired using quadrupole isolation mode and higher-energy collisional dissociation activation type in the ion-trap. The isolation window was 1.2 *m*/*z*, collision energy was 30 NCE, the automatic gain control was set at 100%, and maximum injection time was set as dynamic. Other global settings were set to the following: ion source type, NSI; spray voltage, 1800 V; ion transfer tube temperature, 300 °C. Method modification and data collection were performed using Xcalibur software (Thermo Fisher Scientific).

### Data Analysis for Proteomics

Proteomic analysis was performed using Proteome Discoverer 3.1 (PD 3.1, Thermo Fisher Scientific) software, the Nsp1 protein sequence, and SequestHT with Percolator validation. Percolator false discovery rates were set at 0.001 (strict) and 0.01 (relaxed). Peptide false discovery rates were set at 0.001 (strict) and 0.01 (relaxed), with medium confidence and a minimum peptide length of 6. Carbamidomethyl (C) was set as a static modification; oxidation (M) and Co­(III) complex (H, + 296.0809 Da) were set as dynamic modification; acetyl (protein N-term), Met-loss (Protein N-term M), and Met-loss + acetyl (Protein N-term M) set as dynamic N-terminal modifications. Protein abundance normalization was performed relative to total peptide. Differential expression analysis was performed using an in-house python script.

### Computational Methods

All quantum chemical (QM) NMR calculations were performed using Gaussian16.^28^ To investigate the coordination environment of Co­(III)­(acacen) complexes upon peptide binding, a series of plausible axial ligand substitution models were constructed, including retention of both axial ammonia ligands, substitution of a single axial ammonia ligand by an imidazole ligand, and substitution of both axial ammonia ligands. Initial geometries were fully optimized using the BLYP functional and the def2-SVP basis set. Solvent effects were included using integral equation formalism for a polarizable continuum model (IEPCM).^28,29^

Following geometry optimization, ^59^Co NMR shielding constants were computed using the gauge-including atomic orbital (GIAO) method.^30^ NMR calculations employed a long-range corrected hybrid functional LC-ωPBE^31^ and Douglas–Kroll–Hess (DKH) Gaussian basis sets.^31,32^

^59^Co NMR chemical shifts (δ^59^Co) were calculated using the following equation:

δCo59=σref−σcalc

where σ_ref_ is the calculated shielding constant for [Co­(CN)_6_]^3–^ as reference and σ_calc_ is the calculated shielding constant for each Co­(III)­(acacen) complex in this study.

### Functional Assay

Modified Thermo Scientific 1-Step Human Coupled IVT Kit – DNA (Catalog No. 88881) was employed. Samples were prepared according to the manufacturer’s protocol for GFP expression, followed by addition of test compounds.

Reagents were added to a sterile black wall, clear bottom 96 well plate in the following order using sterile pipet tips: HeLa Lysate (25 μL), Accessory Proteins (5 μL), Reaction Mix (10 μL), pCFE-GFP DNA (0.5 μg/μL, 4 μL), the test compound or control, and nuclease-free water to a final volume of 50 μL.

For Nsp1 inhibition studies, Nsp1 (from previously frozen stocks) was added to obtain a concentration of 1 μM for each reaction. Co­(III)­(acacen)­(NH_3_)_2_^+^ concentrations were either 1 or 5 μM in the final reaction. These samples were first incubated for 24 h at 4 °C, shaking at 80 rpm before being added to the assay. Co­(III)­(acacen)­(NH_3_)_2_^+^ does not inhibit GFP synthesis; this control with a single time point of the assay measured 6 h after incubating at 30 °C can be found in the data repository under associated content (Co­(acacen)_IVT.png).

A SpectraMax iD5Multi-Mode Microplate Reader was used in all experiments. Reaction solutions (50 uL) were placed in a black wall, clear bottom 96 well plate. Samples at 30 °C were shaken for 6 h with time points collected every 10 min. Sample was excitated at 488 nm and monitored at 512 nm.

## Results and Discussion

### Reaction of Co­(III)­(acacen)­(NH_3_)_2_^+^ with Nsp1-CT_10_

We first established the spectroscopic signature of Co­(III)­(acacen)­(NH_3_)_2_^+^ and characterized the kinetics of the reaction with Nsp1-CT_10_ by UV–vis absorption spectroscopy, before probing the coordination environment in detail by nuclear magnetic resonance (NMR) and computational methods. The UV–vis absorption spectrum of Co­(III)­(acacen)­(NH_3_)_2_^+^ displays a characteristic feature at approximately 340 nm, consistent with a low-spin Co­(III) complex comprised of equatorial N_2_O_2_ acacen coordination along with two N-donor axial ammines (Figure 2A). The reaction between Nsp1-CT_10_ and Co­(III)­(acacen)­(NH_3_)_2_^+^ was monitored for 24 h by absorption changes at 340 nm (Figure 2B). Prior literature suggests that this reaction occurs via multistep processes involving sequential aquation and ligand substitution.^33^

**Figure 2:**
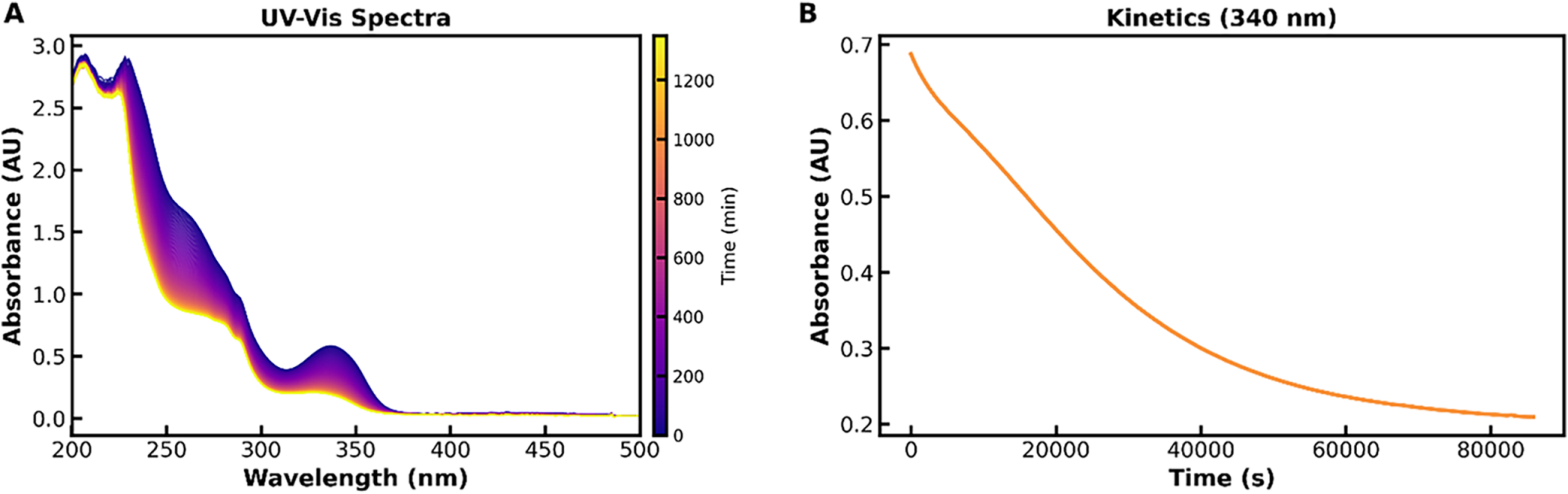
UV–vis spectra and kinetics of the reaction of Co­(III)­(acacen)­(NH_3_)_2_^+^ with Nsp1-CT_10_ in D_2_O. (A) Time evolution of the UV–vis absorption spectrum of Co­(acacen)­(NH_3_)_2_^+^ (100 μM) in aqueous solution with Nsp1-CT_10_ (100 μM). (B) Absorbance decay monitored at 340 nm over the full experimental time course (∼86,000 s), owing to formation of the Nsp1-CT_10_–Co­(acacen) complex.

### ^1^H NMR Spectra

The ^1^H NMR spectrum of Nsp1-CT_10_ in the aromatic region (δ 7–9 ppm) exhibits resonances characteristic of the His165 imidazole and the Trp161 indole groups (Figure 3). A singlet at δ 8.5 ppm likely arises from C(2)-H of the His165 imidazole. The C(5)-H should be near δ 7.4 ppm but is obscured by resonance of the Trp161 indole protons. The C(2)-H chemical shift indicates a substantial imidazolium population.^34^ The ^1^H NMR spectrum of Nsp1-CT_10_ in the presence of Co­(III)­(acacen)­(NH_3_)_2_^+^ provides clear evidence for two complexes in a ratio of about 2.4:1. Resonances at δ 2.19 and 2.35 ppm are assigned to the two pairs of equivalent methyl groups on the acacen ligand in the major complex. Consequently, their integrated areas are defined as 6 protons and serve as the integration reference throughout. A smaller feature at δ 2.25 ppm integrates to 2.5H and is attributable to one pair of equivalent methyl groups in the minor complex. The C(2)-H resonance of the free peptide is all but eliminated in the presence of Co­(III)­(acacen)­(NH_3_)_2_^+^, owing to His165 Co­(III) coordination (Figure 3). Expansion of the 6.0–7.1 ppm region revealed four resonances at δ 6.06 (0.9H), 6.41 (0.3H), 6.98 (1.0H) and 7.3 (0.4H) ppm, none of which was present in the spectrum of free Co­(III)­(acacen)­(NH_3_)_2_^+^ or Nsp1-CT_10_, confirming that they arise from the His165 imidazole of Nsp1-CT_10_ upon coordination to the cobalt center. The resonance at δ 6.98 ppm integrates to approximately 1H relative to the 6H acacen methyl reference and likely arises from the Co­(III)-coordinated imidazole C(2)-H in the major complex. The C(5)-H resonance in Co­(III) imidazole complexes is generally 1 ppm upfield from the C(2)-H resonance,^33,35^ thereby implicating the feature at δ 6.06 ppm as C(5)-H in the major Co­(III) complex. The two remaining resonances (δ 6.41 (0.3H), 7.3 (0.4H)) are attributable to C(5)-H and C(2)-H, respectively, in the minor complex. As Nsp1-CT_10_ contains only one histidine residue, bis-axial imidazole coordination would be expected to yield 2H at this chemical shift, however, the observation of 1H instead supports monoaxial histidine coordination as the predominant binding mode in solution, consistent with Co­(III)­(acacen)­(Im_His165_)­(NH_3_)^+^ as the major species. The minor component could be Co­(III)­(acacen)­(Im_His165_)­(OH_2_)^+^.

**Figure 3:**
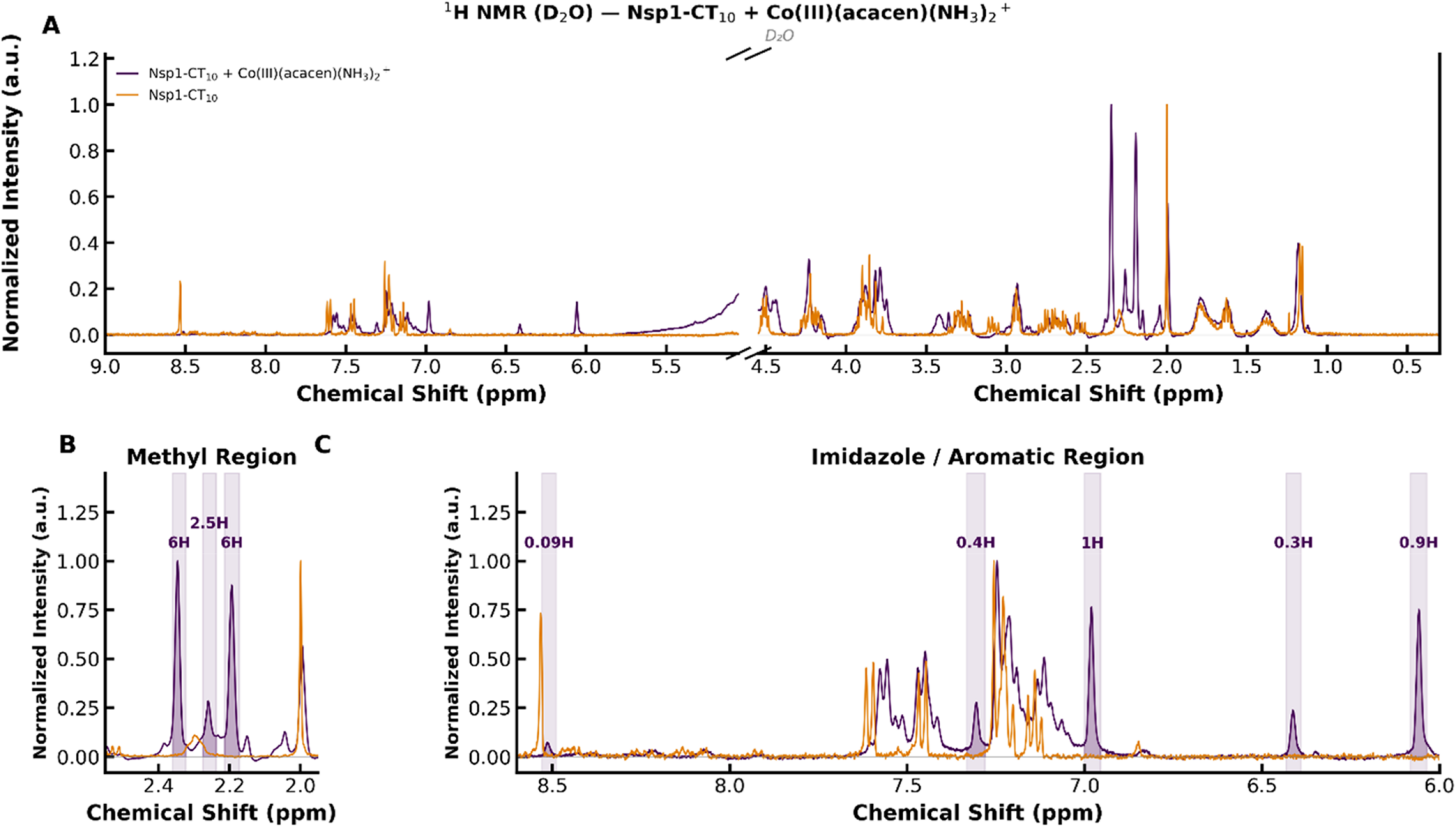
^1^H NMR spectra of Nsp1-CT_10_ before (gold) and after (purple) incubation (24 h) with Co­(III)­(acacen)­(NH_3_)_2_^+^ in D_2_O. (A) Full presaturation ^1^H NMR spectrum (400 MHz, D_2_O) of Nsp1-CT_10_ in the presence of equimolar Co­(III)­(acacen)­(NH_3_)_2_^+^ (10 mM), with the D_2_O solvent peak omitted for clarity. (B) Expanded methyl region (1.95–2.50 ppm) showing two integrated resonances of 6H each (δ 2.323–2.360 ppm and δ 2.172–2.214 ppm), consistent with the two pairs of equivalent methyl groups of the acacen ligand. A smaller feature at δ 2.25 ppm is attributable to a one pair of equivalent acacen methyl protons on a minor population of the peptide coordinated Co­(III) complex. (C) Expanded imidazole and aromatic region (6.00–7.80 ppm) with highlighted resonances attributed to the histidine imidazole of Nsp1-CT_10_ upon coordination to Co­(III). Resonances integrating to 0.88H (δ 6.035–6.082 ppm), and 1H (δ 6.955–7.001 ppm) are assigned to Co­(III)-coordinated His165 imidazole C(5)-H and C(2)-H ring protons in the major complex. The corresponding resonances in the minor complex appear at δ 6.4 and 7.3 ppm. These features are not present in the spectrum of free Co­(III)­(acacen)­(NH_3_)_2_^+^. All integration values are reported relative to the acacen methyl resonances in panel B (6H reference).

### ^59^Co NMR Spectra

Low-spin cobalt­(III) complexes are well suited for investigations by ^59^Co NMR spectroscopy. Cobalt-59 possesses a nuclear spin of I = 7/2 and a quadrupole moment of 0.4 × 10^–24^ cm^2^, thereby enabling NMR monitoring of changes in Co­(III) ligation. ^59^Co line widths are highly sensitive to electric field gradients (EFGs) at the cobalt center and, in turn, to the symmetry of inner-sphere coordination. A more asymmetric coordination produces a larger EFG, accelerating the quadrupolar relaxation and broadening the resonance.^26,36^ Since ^59^Co chemical shifts span a very wide range, approximately 18,000 ppm, they provide exceptional sensitivity to changes in cobalt­(III) coordination.^37–39^ Importantly, line width changes upon ligand substitution or bimolecular binding reflect two distinct contributions: changes in quadrupolar coupling arising from altered coordination symmetry, and changes in the rotational correlation time (τ_c_) of the complex, the latter increasing substantially upon binding to a large biomolecule such as a peptide or protein.^25,26,40^ Both effects manifest as line width broadening and are therefore informative reporters of binding. Of relevance here is that ^59^Co NMR is a powerful tool for monitoring ligand substitution as well as other structural changes in cobalt­(III) Schiff base complexes.

Oxygen-donor ligation is associated with downfield ^59^Co-chemical shifts, owing in part to paramagnetic contributions from low-lying electronic excited states. In contrast, upfield ^59^Co resonance shifts are observed for complexes with N-donor coordination. These findings mean that ^59^Co chemical shifts offer a sensitive readout of axial ligand identity.

The ^59^Co NMR spectrum of Co­(acacen)­(NH_3_)_2_^+^ in D_2_O displays a well-defined resonance at 8472 ppm (Figure 4), consistent with a Co­(III) center in a mixed N_2_O_2_ equatorial environment with axial nitrogen donors. The spectrum was acquired on a 400 MHz instrument, corresponding to an observation frequency of 94.9 MHz for ^59^Co. Lorentzian line shape fitting yielded a peak center of 8472 ppm and a fwhm of 30.85 ppm, corresponding to 2927.6 Hz at this field strength. Since ^59^Co (I = 7/2) is a quadrupolar nucleus, transverse relaxation occurs predominantly through the quadrupolar mechanism, and the line width directly reports on the spin–spin relaxation time T_2_ via T_2_ = 1/(πΔν), giving T_2_ = 0.109 ms.^40–43^ The relatively narrow line width reflects a small electric field gradient at the Cobalt­(III) nuclei, consistent with the well-defined and nearly symmetric coordination geometry imposed by the acacen ligand framework.

**Figure 4:**
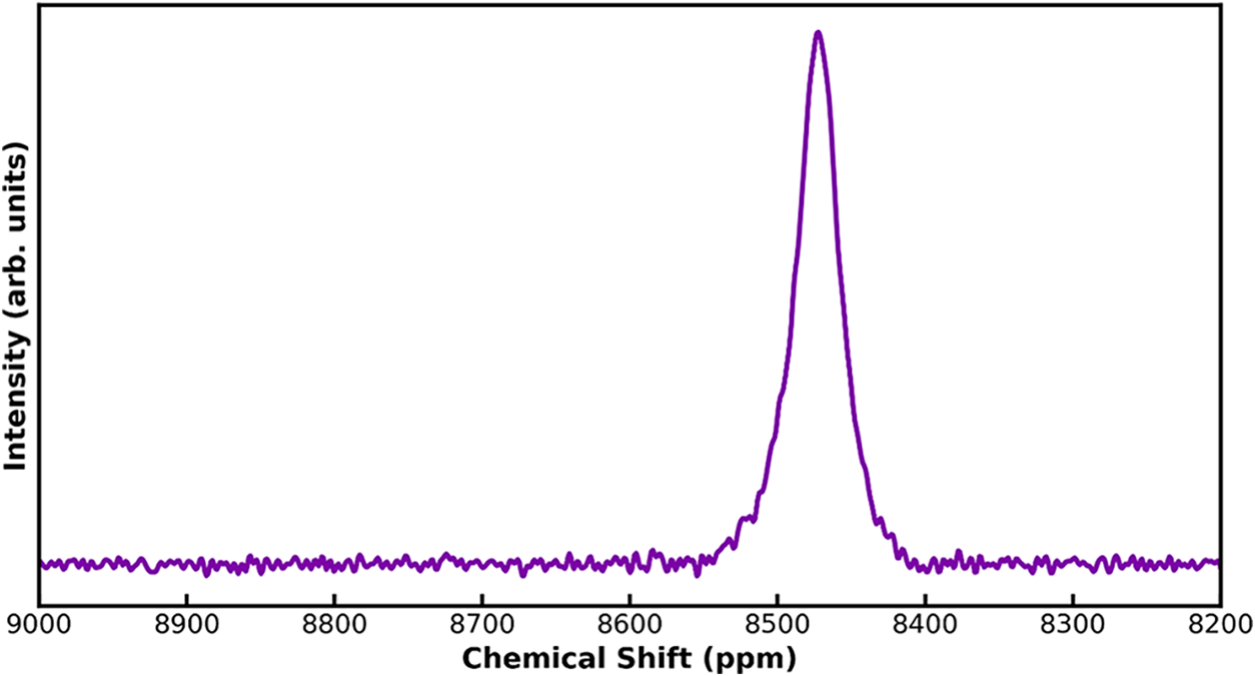
^59^Co NMR spectrum of Co­(III)­(acacen)­(NH_3_)_2_^+^ in D_2_O. The prominent peak is at 8472 ppm.

Following overnight incubation of 1:1 Co­(III)­(acacen)­(NH_3_)_2_^+^:Nsp1-CT_10_, the ^59^Co NMR peak assigned to Co­(III)­(acacen)­(NH_3_)_2_^+^ was replaced by a new peak at 8796 ppm (Figure 5). Lorentzian line shape fitting yielded a peak fwhm of 20.69 ppm, corresponding to 1963.5 Hz at this field strength, consistent with T_2_ = 0.162 ms. Our ^1^H NMR measurements provide compelling evidence that under these conditions Co­(III)­(acacen)­(Im_His165_)­(NH_3_)^+^ and possibly Co­(III)­(acacen)­(Im_His165_)­(OH_2_)^+^ are formed. Calculated ^59^Co chemical shifts support this assignment of the 8796 peak to Co­(III)­(acacen)­(Im_His165_)­(NH_3_)^+^ (*vide infra*).

**Figure 5:**
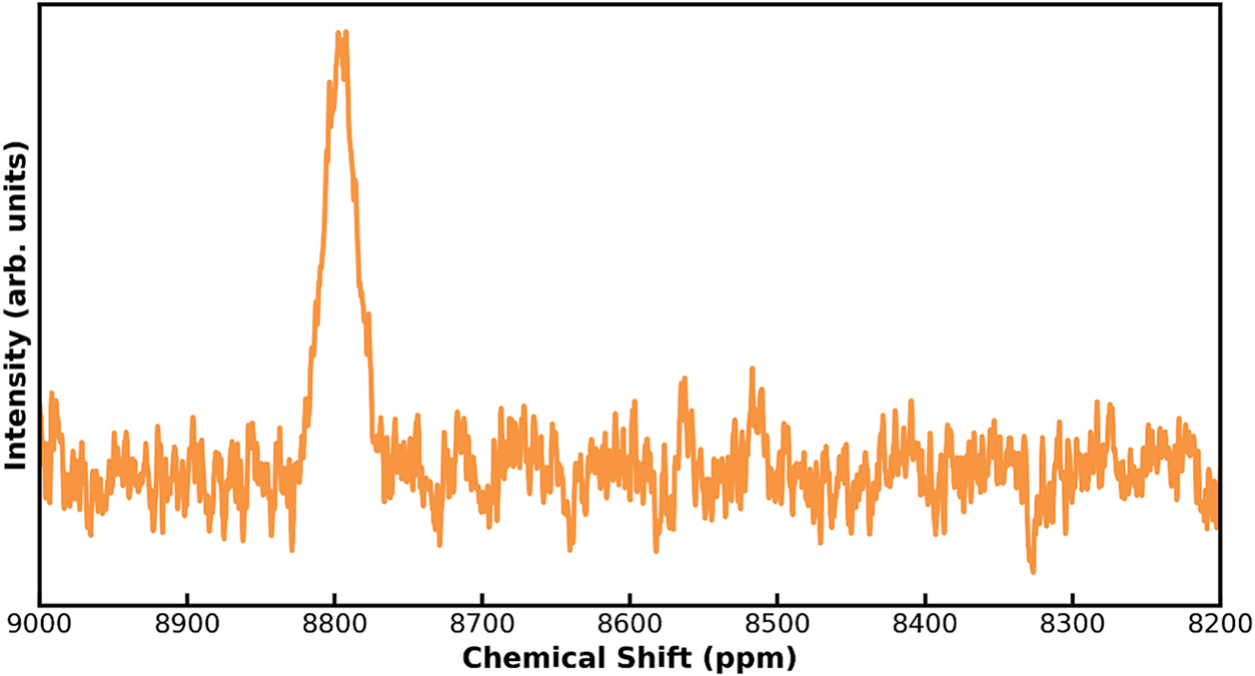
^59^Co NMR spectrum of Co­(III)­(acacen)­(NH_3_)_2_^+^ after 36-h incubation with 1 equiv of Nsp1-CT_10_ (the spectrum was collected in D_2_O; the prominent peak is at 8796 ppm).

The 33% reduction in ^59^Co NMR line width for Co­(III)­(acacen)­(Im_His165_)­(NH_3_)^+^ relative to Co­(III)­(acacen)­(NH_3_)_2_^+^ is somewhat unexpected based on its lower symmetry and larger size, both of which can contribute to line broadening. Systematic investigations of ^59^Co NMR relaxation times in six-coordinate Co­(III) complexes suggest that line widths are dominated by the quadrupolar relaxation mechanism.^42^ The primary contributors to this relaxation are the asymmetry of the electric field gradient tensor (efg), the quadrupolar coupling constant, and the rotational correlation time of the complex. The study of 12 diverse Co­(III) complexes revealed that line widths and, hence, relaxation rates correlate well with quadrupolar coupling constants.^42^ The symmetry and size of the complexes were less reliable indicators of ^59^Co NMR line widths. The line narrowing in Co­(III)­(acacen)­(Im_His165_)­(NH_3_)^+^, then, can be attributed to a modest reduction in quadrupolar coupling constant, owing to a decrease in the principal efg component.

### Computational Analysis

An important question that arises from these observations is whether peptide coordination involves substitution of one or both axial ligands of Co­(III)­(acacen)­(NH_3_)_2_^+^, and, if only one ligand is displaced, the identity of the remaining ligand. To elucidate the nature of this coordination change, QM calculations were performed for a series of plausible axial ligand combinations at the Co­(III) center, including water, ammonia, and imidazole ligands (Figure 6 and Table 1). The calculated ^59^Co NMR chemical shifts span a wide range (8500–12,500 ppm), highlighting the sensitivity of these chemical shifts to the nature of axial ligation.

**Figure 6:**
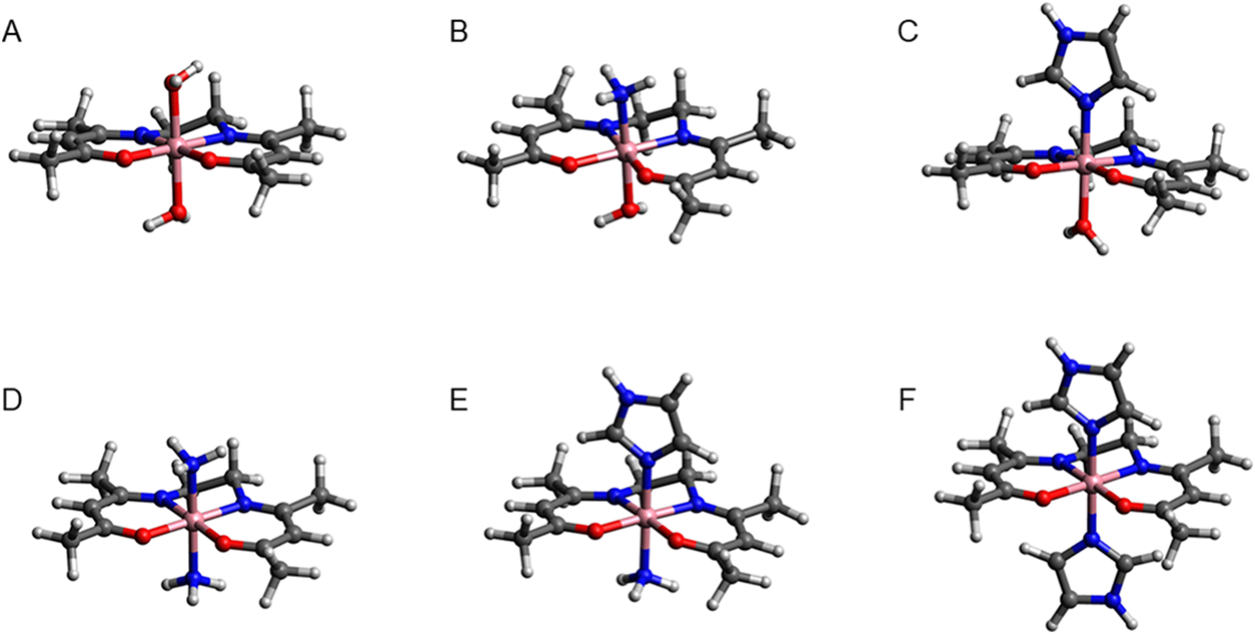
Computational structures of Co­(III)­(acacen) complexes with varying axial ligand combinations. All structures share the same equatorial N_2_O_2_ coordination environment defined by the acacen Schiff base ligand, with cobalt shown in pink, nitrogen in blue, oxygen in red, carbon in gray, and hydrogen in white. (A) Co­(acacen)­(H_2_O)_2_^+^, with two axial water ligands. (B) Co­(acacen)­(H_2_O)­(NH_3_)^+^, with mixed axial water and ammonia coordination. (C) Co­(acacen)­(H_2_O)­(Im)^+^, with axial water and imidazole ligands. (D) Co­(acacen)­(NH_3_)_2_^+^, with two axial ammonia ligands. (E) Co­(acacen)­(NH_3_)­(Im)^+^, with mixed axial ammonia and imidazole coordination. (F) Co­(acacen)­(Im)_2_^+^, with two axial imidazole ligands. The series illustrates the progressive substitution of axial ligands from oxygen donors (H_2_O) to nitrogen donors (NH_3_ and imidazole), which is relevant to the coordination chemistry of Co­(III)­(acacen) in the presence of histidine-containing peptides such as Nsp1-CT_10_.

**Table 1:** Computationally Predicted ^59^Co NMR [Co­(III)­(acacen)] Complex Chemical Shifts with Variations in Axial Ligation

Co(III)(acacen) complex	axial ligands	predicted NMR chemical shift (ppm)
**A**	H_2_O–H_2_O	12,493
**B**	H_2_O-NH_3_	9772
**C**	H_2_O-imidazole	10,251
**D**	NH_3_–NH_3_	8505
**E**	NH_3_–imidazole	8752
**F**	imidazole–imidazole	9240

The calculated chemical shift for NH_3_–NH_3_ axial ligation is 8505 ppm, in excellent agreement with the experimentally observed 8477 ppm for Co­(III)­(acacen)­(NH_3_)_2_^+^. This agreement supports the reliability of the computational approach in capturing the electronic environment at the Co­(III) center, thereby providing a validated reference point for assigning ligand substitutions.

Of interest, the calculated ^59^Co chemical shift of 8752 ppm for NH_3_–imidazole axial ligation (Figure 5E) is near the experimentally observed 8877 ppm peak for the Co­(III) derivative of Nsp1-CT_10_. In contrast, models involving substitution of both axial ammines (imidazole–imidazole, 9240 ppm) or coordination by water ligands (H_2_O–NH_3_, H_2_O–imidazole, and H_2_O–H_2_O) have calculated chemical shifts that deviate substantially from the experimental NMR peak position. Based on these data, we conclude that reaction of Co­(III)­(acacen)­(NH_3_)_2_^+^ with Nsp1-CT_10_ involves displacement of an axial ammine by the His165 imidazole.

### Fragmentation and Mass Spectrometry Analysis

Axial ligand substitutions in Co­(III)­(acacen)­(NH_3_)_2_^+^ will not be restricted to His165 in full-length Nsp1, as the protein contains seven histidine residues: His13, His45, His81, His83, His110, His134, and His165 (Figure 7). Of these, His13, His45, His81, His83, and His110 reside within the structured N-terminal domain (residues 1–127), His134 is in the linker region, and His165 is in the disordered C-terminal domain. These assignments are based on the NMR solution structure of Nsp1 (PDB ID: 8AOU).^44^ In this structure His45, His81, His83, His134, and His165 are surface exposed and therefore accessible to metal complexes, whereas His13 and His110 are buried within the globular core of the N-terminal domain. We expect Co­(III)­(acacen)­(NH_3_)_2_^+^ to bind to the accessible histidine residues in the protein.^45^

**Figure 7:**
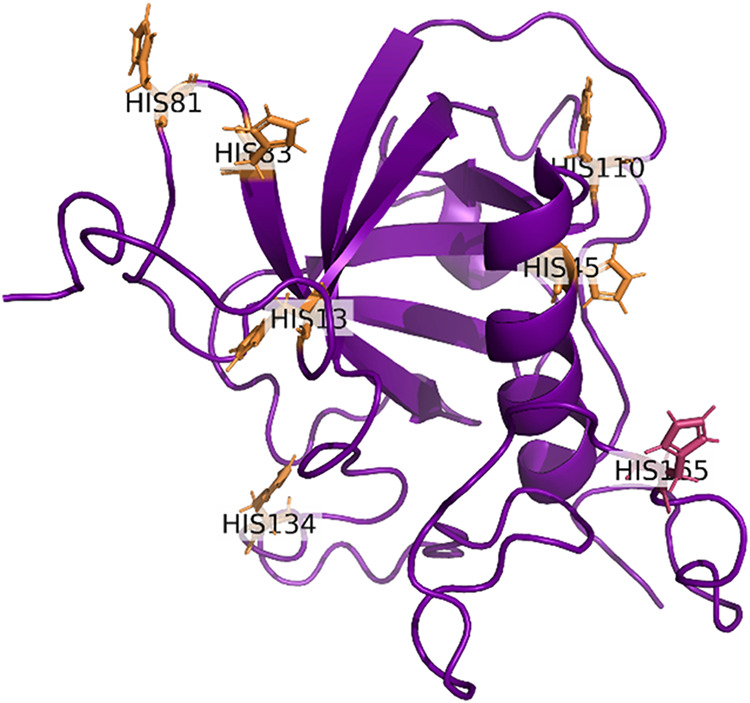
NMR solution structure of full-length Nsp1, with the 7 histidine residues highlighted. PDB ID: 8AOU.

Recombinantly expressed Nsp1 was incubated with one equivalent of Co­(III)­(acacen)­(NH_3_)_2_^+^ prior to proteolytic fragmentation. Trypsin digestion yielded peptide fragments that in most cases contained a single histidine residue (the exception being the closely spaced His81 and His83 residues, which were not found in separate fragments). The resulting peptide mixtures were analyzed by mass spectrometry to determine which fragments contained Co­(III) complexes. Three sites of Co­(III) coordination were identified: His13, the His81/His83 region, and His165.

### Functional Assay

An *in vitro* translation assay was employed in which protein synthesis was monitored by production of green fluorescent protein (GFP). In this assay, recombinantly expressed full-length Nsp1 produced in *E. coli* robustly suppresses translation, resulting in decreased GFP expression. Since His165 in full-length Nsp1 binds to Co­(III)­(acacen)­(NH_3_)_2_^+^, we asked whether Co­(III) coordination might be sufficient to disrupt Nsp1’s interaction with the 40S ribosome and thereby restore GFP production.

However, we found that treatment of Nsp1 with Co­(III)­(acacen)­(NH_3_)_2_^+^ did not restore GFP expression (Figure 8). Although full-length Nsp1 inhibited translation relative to the control, the incubation with either 1 or 5 equiv of Co­(III)­(acacen)­(NH_3_)_2_^+^ (relative to Nsp1 concentration) failed to rescue GFP production.

**Figure 8:**
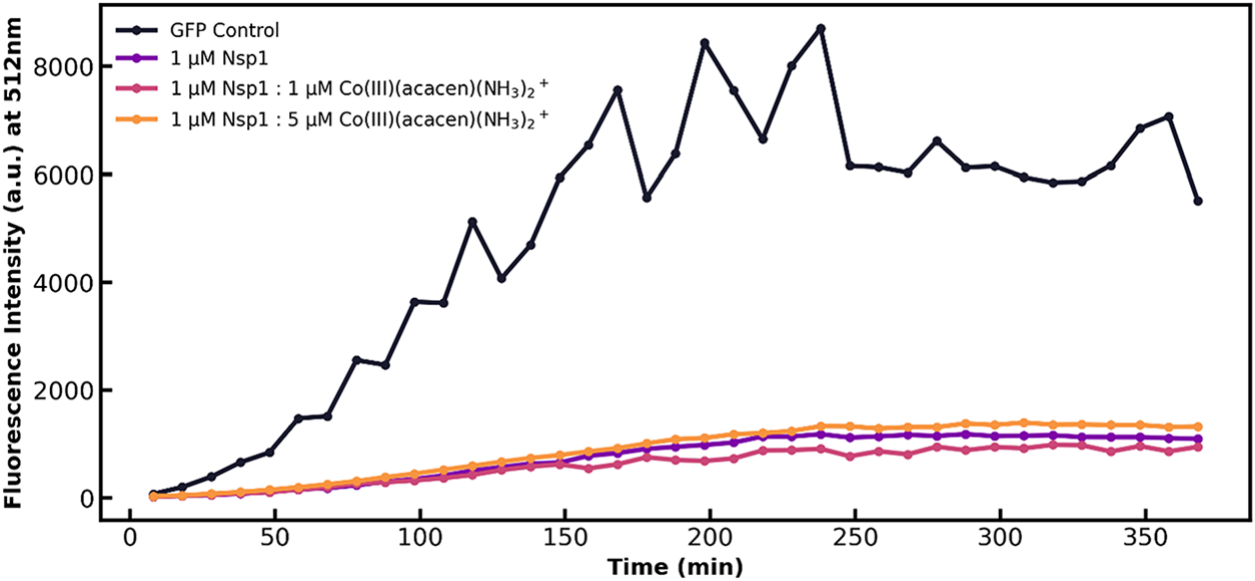
Our *in vitro* translation assay confirmed GFP is not produced upon incubation of full-length Nsp1 with Co­(III)­(acacen)­(NH_3_)_2_^+^.

## Concluding Remarks

Co­(III)­(acacen)­(NH_3_)_2_^+^ reacts with full-length Nsp1, and mass spectrometric analysis of protein fragments revealed multiple Co­(III) coordination sites: His13, the His 81/His83 region, and His165. Functional assays, however, demonstrate that targeting Nsp1 with Co­(III)­(acacen)­(NH_3_)_2_^+^ does not restore protein translation. In sharp contrast, several studies have shown that a Lys164Ala/His165Ala double mutation restores protein translation,^4,5,8^ thereby indicating a critical role for both residues in Nsp1 binding to the 40S ribosome. Of importance is work on Nsp1 single-site mutants strongly indicating Lys164 is the key residue for inhibition of host protein synthesis^9^ and that mutation of His165 alone had a substantially smaller impact. The importance of Lys164 suggests that Lys164:His165 bidentate metal-binding would inhibit Nsp1 interactions with the 40S ribosome. It should be noted that Co­(III)­(acacen)­(NH_3_)_2_^+^ acts as a general histidine modifier rather than a targeted inhibitor of His165, as evidenced by the coordination to multiple surface exposed histidines in full length Nsp1. Future efforts directed toward bidentate coordination to a Lys164:His165 motif, or toward Co­(III) complexes with improved selectivity for this functionally critical site, may provide a more effective strategy for disrupting Nsp1-mediated translational suppression.

## Supplementary Material



## Notes

UV–vis kinetics data, ^1^H NMR, and ^59^Co NMR spectra (PDF)
