# Supplementary material for: Cobalt(III)–Schiff Base Coordination to Nsp1, a SARS-CoV‑2 Protein
Source: Inorg Chem. 2026 Jul 22;65(30):17325–33. doi: 10.1021/acs.inorgchem.6c01698 (PMC13439656; doi:10.1021/acs.inorgchem.6c01698)
Supplement: Supplementary file 1 [file ic6c01698_si_001.pdf]

Supporting Information

# Cobalt(III)–Schiff Base Coordination to Nsp1, a SARS-CoV-2 protein

*AUTHOR NAMES Maryann Morales, Moon Young Yang , William A. Goddard III , Harry B. Gray* , and Jay R. Winkler**

AUTHOR ADDRESS Beckman Institute, California Institute of Technology, Pasadena, CA 91125

*Email: hbgray@caltech.edu (Harry Gray)

jwinkler@caltech.edu (Jay Winkler)

**UV-Vis Kinetic Studies**

**Figure S1.** Co(III)(acacen)$(NH_3)_2^+$ Kinetics with $D_2O$………………………………...3

**Figure S2.** Co(III)(acacen)$(NH_3)_2^+$ Kinetics with Imidazole (equilmolar)……………….3

**Figure S3.** Co(III)(acacen)$(NH_3)_2^+$ Kinetics with Imidazole (10 fold excess)……………4

**$^1$H NMR**

**Figure S4.** (acacen) ligand spectrum…………………………………………………...5

**Figure S5.** [Co(III)(acacen)$(NH_3)_2$]Cl spectrum ………………………………………5

**Figure S6.** Nsp1-$CT_{10}$ spectrum…………………………. ……………………………7

**$^{59}$Co NMR**

**Figure S7.** Co(III) Hexamine spectrum………………………...………………………8

## UV- Vis Kinetics

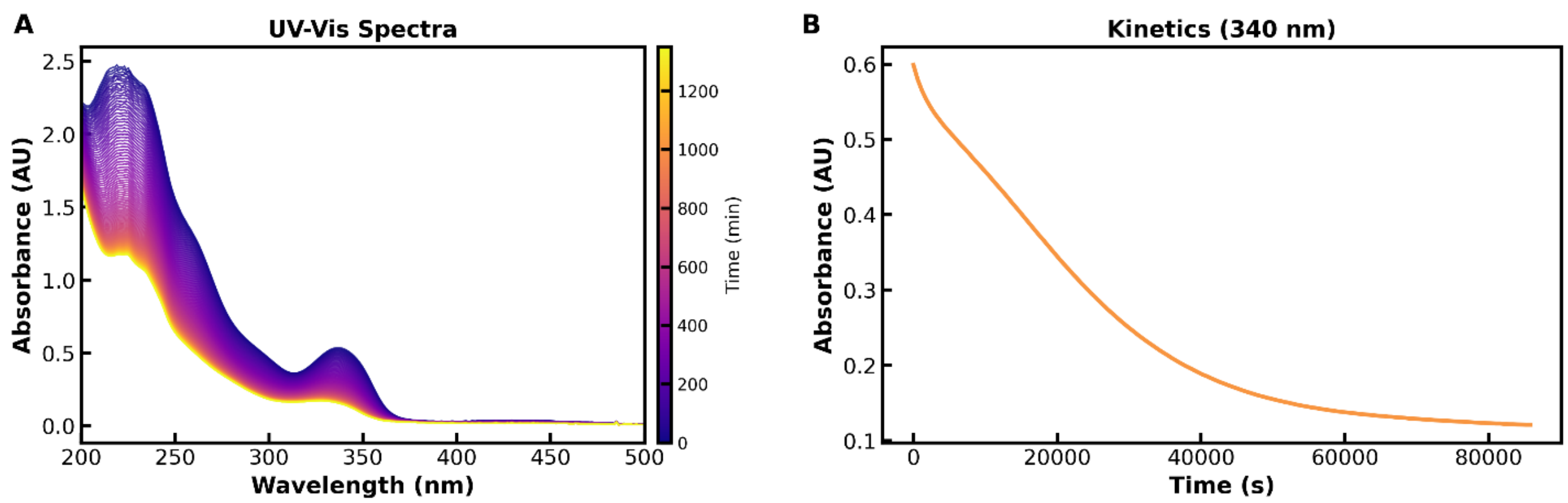


**Figure S1.** Kinetic analysis of Co(III)(acacen)$(NH_3)_2^+$ aquation ($D_2O$) monitored by UV-Vis spectroscopy. **(A)** Time-resolved UV-Vis spectra recorded over 24 hours, showing progressive decrease in absorbance across the 200–500 nm range as the reaction proceeds. **(B)** Absorbance decay at 340 nm over time, reflecting the loss of the starting complex.

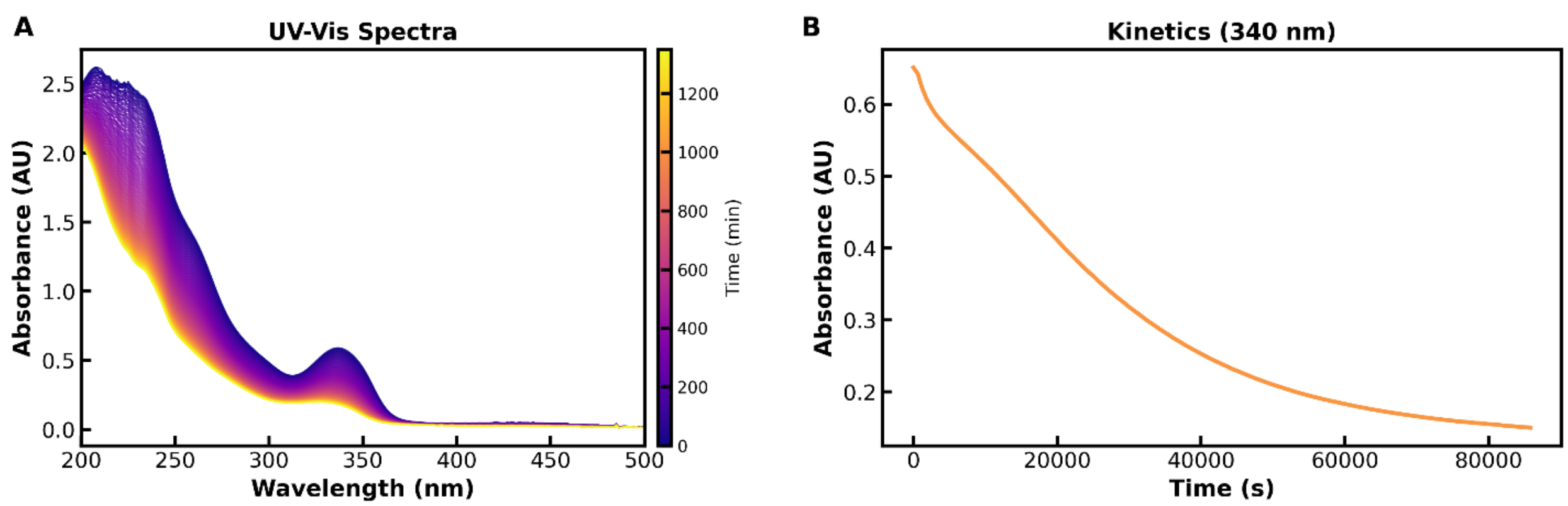


**Figure S2.** Kinetic analysis of Co(III)(acacen)$(NH_3)_2^+$ substitution with equimolar imidazole monitored by UV-Vis spectroscopy. **(A)** Time-resolved UV-Vis spectra recorded over 24 hours, showing decay of absorbance across 200–500 nm alongside the characteristic growth of a band

near 340 nm consistent with imidazole coordination. **(B)** Absorbance decay at 340 nm over 86,400s, reflecting consumption of the starting complex.

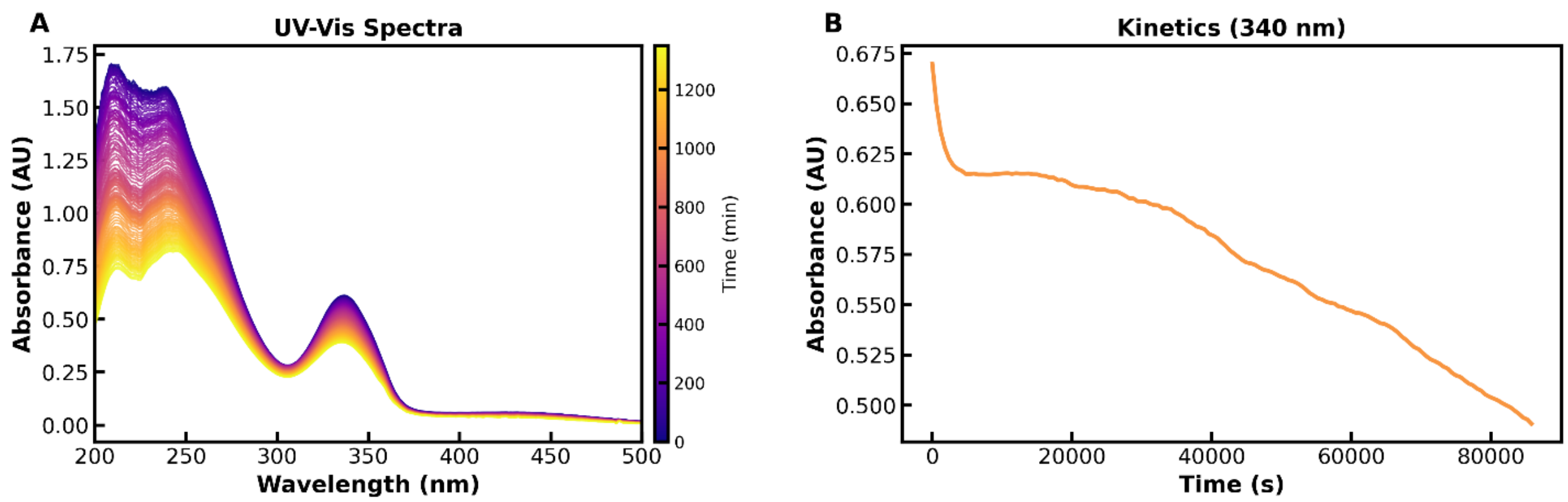


**Figure S3.** Two-phase kinetic analysis of Co(III)(acacen)$(NH_3)_2^+$ reaction with excess imidazole monitored by UV-Vis spectroscopy**. (A)** Time-resolved UV-Vis spectra recorded over 24 hours, showing progressive changes in absorbance across 200–500 nm as the reaction proceeds. **(B)** Absorbance decay at 340 nm over 86,400 s, with shaded regions highlighting the fast (blue) and slow (orange) kinetic phases. Experiments were conducted with [Co(III)(acacen)$(NH_3)_2^+$] = 100 μM and [imidazole] = 1 mM in $D_2O$ at room temperature.

## $^1$H NMR

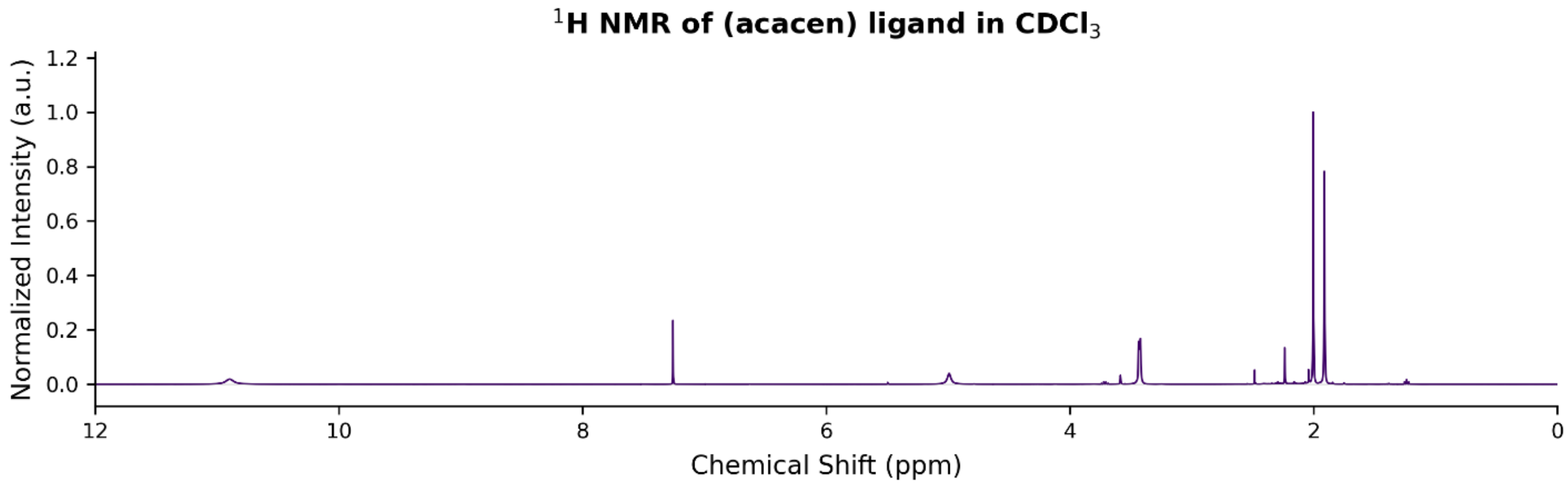


**Figure S4.** $^1$H NMR spectrum of acacen in $CDCl_3$. $^1$H NMR spectrum (400 MHz, $CDCl_3$) of the acacen ligand, confirming the identity of the compound prior to complexation with cobalt. The observed chemical shifts are consistent with the expected proton environments of the acacen scaffold. $^1$H NMR (400 MHz, $CDCl_3$) δ 10.90 (s, 2H), 4.99 (s, 2H), 3.49 – 3.36 (m, 4H), 2.00 (s, 6H), 1.91 (s, 6H).

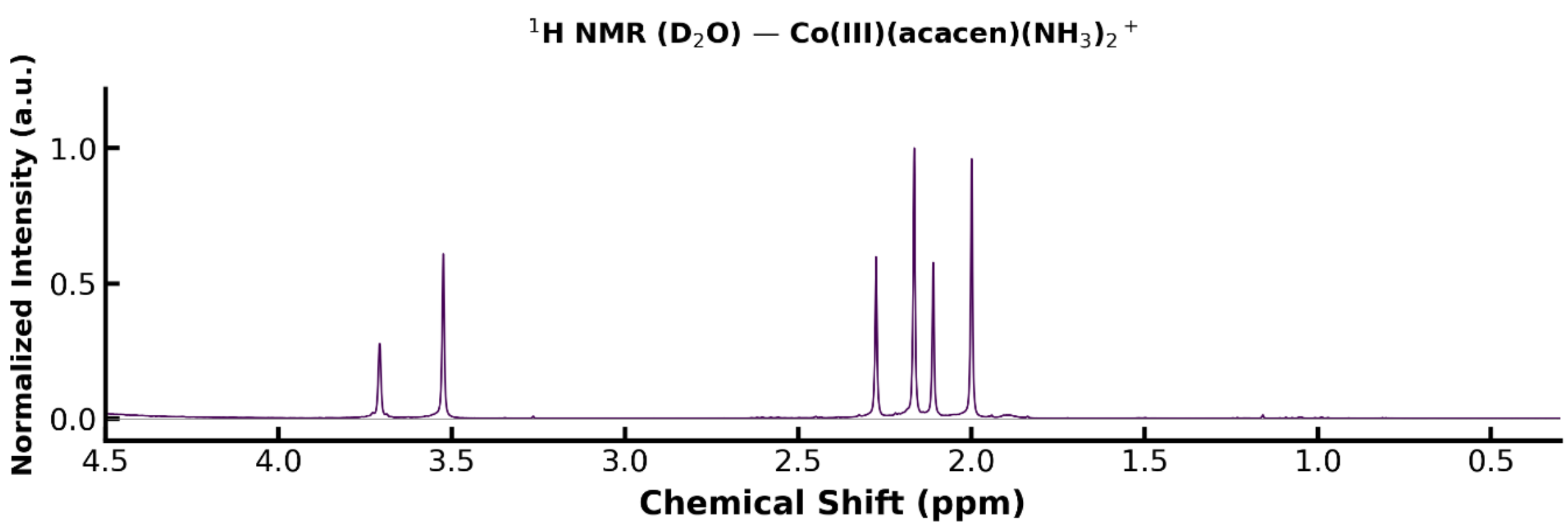


**Figure S5.** $^1$H NMR spectrum of $Co(III)(acacen)(NH_3)_2^+$ in $D_2O$. $^1$H NMR spectrum (400 MHz, $D_2O$) of $Co(III)(acacen)(NH_3)_2^+$ (10 mM). The spectrum reveals the presence of two species in solution: the bis-ammine complex $Co(III)(acacen)(NH_3)_2^+$ and the mono-aquated species $Co(III)(acacen)(NH_3)(OD_2)^+$, arising from partial axial ligand exchange in deuterated solvent.

Resonances at δ 3.52 (s, 4H), 2.17 (s, 6H), and 2.00 (s, 6H) are assigned to $Co(III)(acacen)(NH_3)_2^+$, while peaks at δ 3.71 (s, 2H), 2.28 (s, 4H), and 2.11 (s, 4H) are attributed to the $Co(III)(acacen)(NH_3)(OD_2)^+$ species. Full $^1H$ NMR: δ 3.71 (s, 2H), 3.52 (s, 4H), 2.28 (s, 4H), 2.17 (s, 6H), 2.11 (s, 4H), 2.00 (s, 6H). The relative populations of the two species were estimated by normalizing the integration of each resolved resonance to the number of protons it represents and averaging across all peaks assigned to each species. This analysis yielded per-molecule integration values of 0.97 for $Co(III)(acacen)(NH_3)_2^+$ (averaged over δ 3.52, 2.17, and 2.00 ppm) and 0.97 for $Co(III)(acacen)(NH_3)(OD_2)^+$ (averaged over δ 3.71, 2.28, and 2.11 ppm), corresponding to an approximately 1:1 ratio of the two species in solution. This is consistent with partial aquation of one axial ammine ligand under the experimental conditions used.

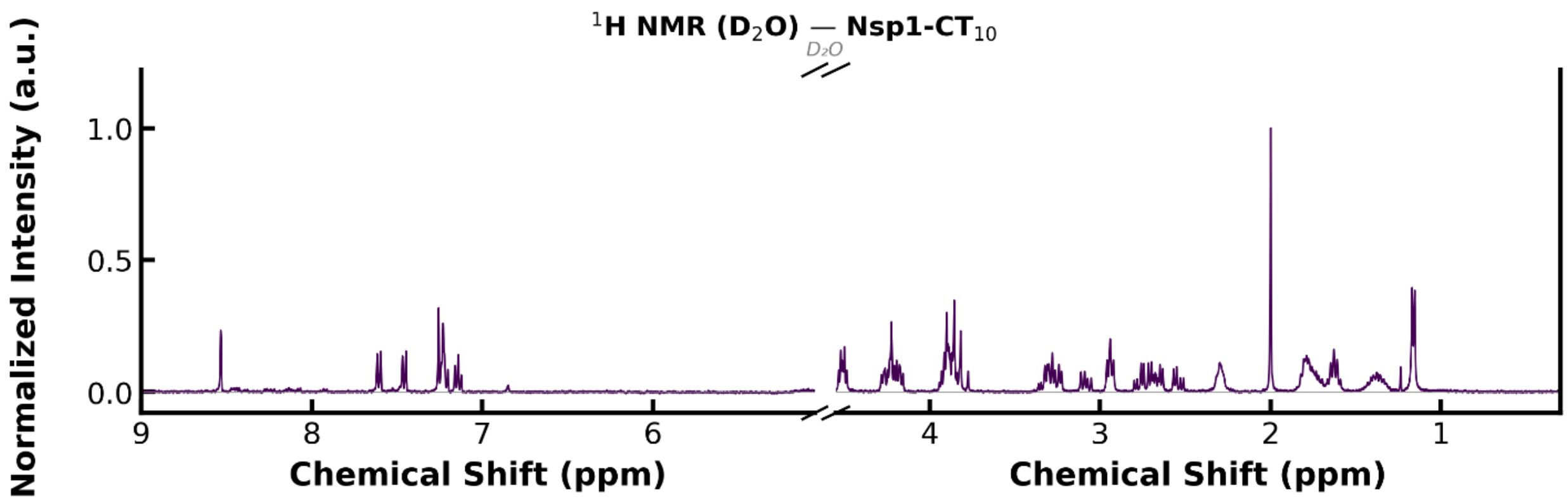


**Figure S6.** Presaturation $^1$H NMR spectra (400 MHz, $D_2O$) of Nsp1-$CT_{10}$ at 10 mM.The $D_2O$ solvent peak (δ ~4.79 ppm) has been omitted for clarity.

## 59Co NMR

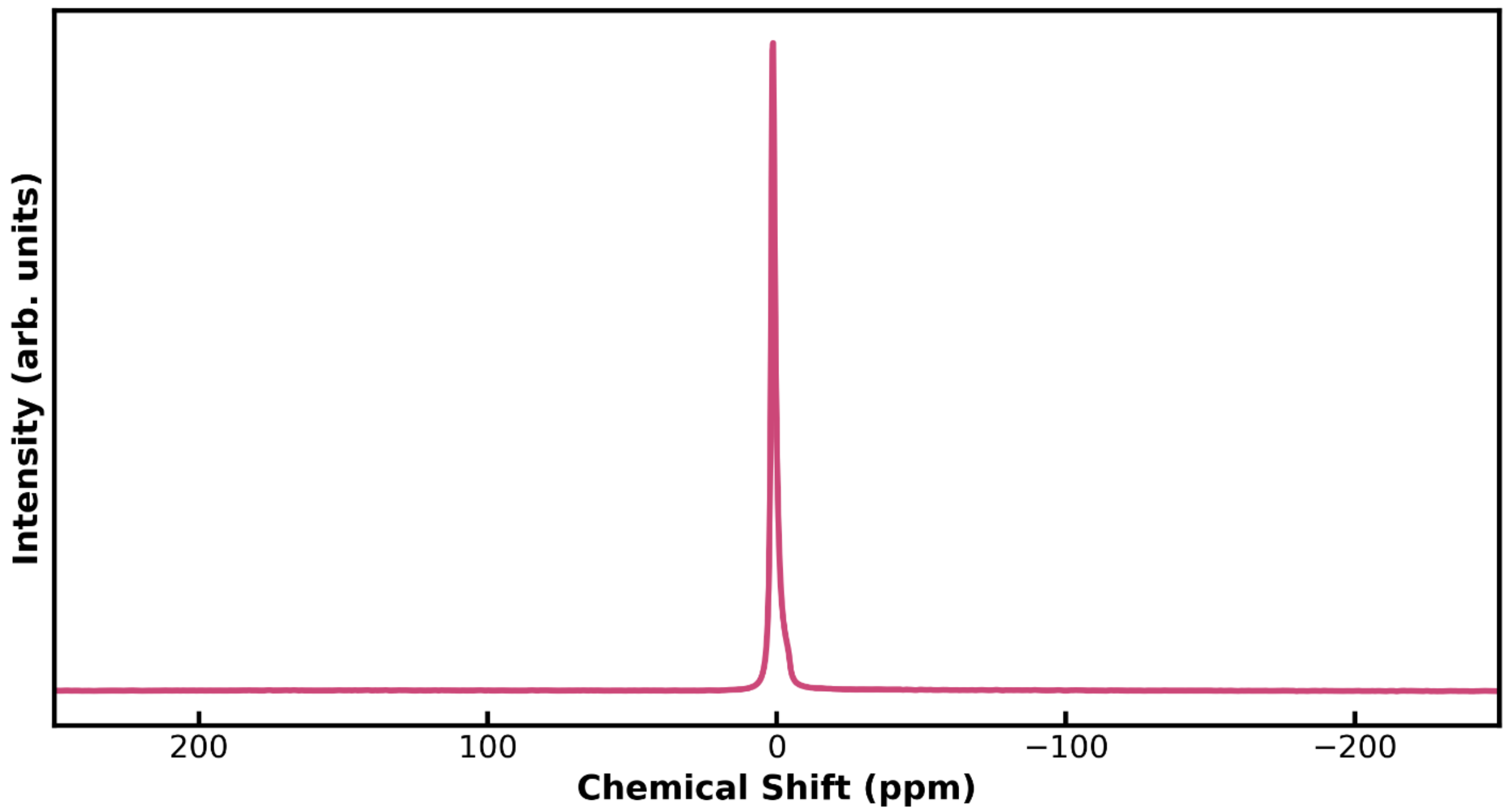


**Figure S7.** $^{59}$Co NMR spectrum of $K_3[Co(CN)_6]$ in $D_2O$. $^{59}$Co NMR spectrum (400 MHz, $D_2O$) of $K_3[Co(CN)_6]$ (100 mM), used as an external chemical shift reference standard ($\delta = 0$ ppm) for $^{59}$Co NMR measurements. The sharp, well-resolved signal confirms the integrity of the reference sample and is consistent with the highly symmetric octahedral coordination environment of the hexacyanocobaltate(III) anion, which minimizes quadrupolar relaxation and yields a narrow linewidth.
